# 

**Published:** 1891-06

**Authors:** 


					﻿jTjisrE, i89i.
OLD SERIES .
Vol. xxv
& 1855
jfe ^fawFSK
SedeM^O
^JOURNAk

l\J H. V. M. MILLER, M. D., LL D
/ VIRGIL 0. HARDON, M D ’
F. W. McRAE, M. D
L.	P. KENNEDY, A. M., M D
M.	B. HUTCHINS, M. D
L. B. GRANDY, M. D
t®UBUSHED By James p.harrison&c go
MP	__ SgTLAHTA.GA.
, at the poat.,(ffice af^^®°^,3®*li *2-<>O A YKAfL
				

